# Power Line Segmentation Algorithm Based on Lightweight Network and Residue-like Cross-Layer Feature Fusion

**DOI:** 10.3390/s25113551

**Published:** 2025-06-04

**Authors:** Wenqiang Zhu, Huarong Ding, Gujing Han, Wei Wang, Minlong Li, Liang Qin

**Affiliations:** 1 School of Electronic and Electrical Engineering, Wuhan Textile University, Wuhan 430200, China; zhuwq@wtu.edu.cn (W.Z.); 2215363017@mail.wtu.edu.cn (H.D.); 2215363043@mail.wtu.edu.cn (W.W.); 2215363032@mail.wtu.edu.cn (M.L.); 2 School of Electrical Engineering and Automation, Wuhan University, Wuhan 430072, China; qinliang@whu.edu.cn

**Keywords:** power line segmentation, lightweight UNet, Ghost Module, class residual addition

## Abstract

Power line segmentation plays a critical role in ensuring the safety of transmission line UAV inspection flights. To address the challenges of small target scale, complex backgrounds, and excessive model parameters in existing deep learning-based power line segmentation algorithms, this paper introduces RGS-UNet, a lightweight segmentation model integrating a residual-like cross-layer feature fusion module. First, ResNet18 is adopted to reconstruct a UNet backbone network as an encoder module to enhance the network’s feature extraction capability for small targets. Second, ordinary convolution in the residual block of ResNet18 is optimized by introducing the Ghost Module, which significantly reduces the computational load of the model’s backbone network. Third, a residual-like addition method is designed to embed the SIMAM attention mechanism module into both encoder and decoder stages, which improves the model’s ability to extract power lines from complex backgrounds. Finally, the Mish activation function is applied in deep convolutional layers to maintain feature extraction accuracy and mitigate overfitting. Experimental results demonstrate that compared with classical UNet, the optimized network achieves 2.05% and 2.58% improvements in F1-Score and IoU, respectively, while reducing the parameter count to 57.25% of the original model. The algorithm achieves better performance improvements in both accuracy and lightweighting, making it suitable for edge-side deployment.

## 1. Introduction

Regular inspection of power lines is an important guarantee for the safe operation of power systems. The traditional manual inspection is inefficient and unreliable [1,2]. With the increasing maturity of unmanned aerial vehicle (UAV) technology, its replacement of manual inspection has become a development trend [3]. To ensure UAVs accurately avoid power lines and prevent crashes during inspection, a finer target detection of power lines, i.e., power line segmentation (also known as power line extraction), is required [4].

Power line segmentation algorithms are mainly categorized into traditional image processing methods and semantic segmentation methods based on deep learning [5]. Although the traditional image processing algorithm has low complexity, it is only suitable for scenes with simple image backgrounds; it is also greatly affected by lighting conditions and the environment, and the detection accuracy is not high [6,7,8,9,10,11].

Deep learning algorithms based on semantic segmentation convolutional neural networks can realize accurate segmentation of power lines at the pixel level, learn context-aware features, and improve the accuracy of segmentation through end-to-end learning. Currently, deep learning has become the main research method for power line segmentation algorithms. YANG et al. [12] designed a model consisting of residual convolutional neural network (RCNN) branches and recursive RCNN branches and introduced a context fusion block (CFB). The algorithm, although designed with specific modules to deal with the noise in complex backgrounds, still suffers from segmentation due to the similarity of the backgrounds and the power line texture incompleteness. KONG et al. [13] proposed a residual path unit based on the UNet network and introduced an attention fusion block to enhance the target detection capability of the model. Although the segmentation accuracy is improved, the structure is more complex due to the introduction of more residual convolution blocks at jump connections, resulting in parameter redundancy and bringing higher computational costs and storage requirements. Liu Jiawei et al. [14] improved the UNet network and designed four UNet models with different network depths and numbers of convolutional kernels; although the segmentation accuracy in complex contexts is improved, the extraction sense field of the current model is small, and segmentation and extraction is difficult for the case of small proportional sizes of the power line pixels. Yang et al. [15] proposed a multi-scale feature fusion attention fusion module for the insufficient processing of local contextual feature mapping in DCNN, which improves the segmentation accuracy of the algorithm. However, the network model parameters are large and not easy to be deployed on edge devices with limited storage space. HAN et al. [16] have a small number of parameters by replacing the backbone network VGG of UNet with the lightweight GhostNet combined with the SA attention mechanism, but the algorithm has a limited segmentation effect when dealing with the complexity of power line contexts due to the lightweight nature of GhostNet.

In summary, power line segmentation algorithms have made some progress in the field of semantic segmentation-based convolutional neural networks, but for power line images with small target size, complex terrain backgrounds, and large number of parameters in the algorithmic model, the existing algorithms still have a large space for improvement and optimization in terms of recognition accuracy, model size, and so on. This paper proposes a lightweight semantic segmentation network based on UNet, which realizes the high-precision segmentation of power lines. The primary contributions are as follows:

(1) To address the issue of small target size of power lines, ResNet18 is used in the encoder part to reconstruct the original backbone VGG [17], and the skip connection in the residual block of ResNet18 can directly transfer the input information to the later structural layer to increase the depth of the network, which strengthens the ability of extracting shallow information of small-size power lines.

(2) To solve the problem of a large number of model parameters, the ordinary convolution in the ResNet18 residual block of the Ghost Module structural optimization coding module is introduced, which effectively reduces the computational amount of the model while ensuring the detection accuracy.

(3) Aiming at the problem of a complex terrain background in which the power lines are located, a class of the residual cross-layer feature fusion module addition method is designed, and a SIMAM module is introduced in the decoder and encoder with this addition method, which effectively improves the segmentation accuracy of the power lines without increasing the number of any parameter by giving each neuron the corresponding weight energy.

(4) In addition, the Mish activation function is also introduced in the residual block deep convolution, which is more capable of matching and improving the performance of the neural network architecture than the original ReLU function and improves the algorithm’s generalization ability and robustness.

## 2. Materials and Methods

### 2.1. Enhanced Small Size Shallow Feature Extraction Based on Backbone ResNet18

The original UNet model uses VGG as the network backbone, which consists of a stack of multiple convolutional and pooling layers with a total network convolution depth of 19 layers. In general, as the number of network convolution layers increases, the network backbone causes the problem of gradient disappearance during backpropagation. This makes it difficult for the network to learn the effective features of images. Currently, there are two solutions:

(1) Directly select the network backbone with fewer convolutional layers to simplify the network structure and reduce the depth of network convolution; the more typical networks are PP-LCNet [18], MobileNetV3 [19], and GhostNet v2 [20], which all belong to the lightweight neural network backbone.

(2) The ResNet [21] family (including various network models such as ResNet18 [22], ResNet50, ResNet101, etc., where the numbers denote the network layer count) serves as the network backbone. By introducing “residual connections”, this design enables the feature information extracted by the network to skip intermediate layers directly, thereby preserving shallow-layer information in the network architecture.

Although the simplified network structure effectively circumvents the gradient vanishing issue stemming from an excessive number of deep layers, it simultaneously leads to the suboptimal extraction of shallow edge details pertaining to power line features. This limitation is attributed to the network’s relatively modest depth and complexity, which impede comprehensive feature representation.

**Figure 1:**
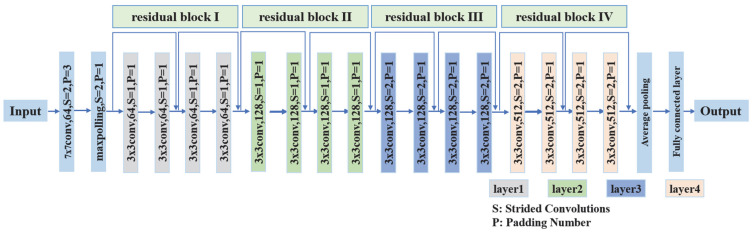
ResNet18 backbone network structure diagram.

In contrast to VGG, which contains more convolutional and denser fully connected layers, the ResNet architecture introduces “residual connections” to strengthen the backbone network for feature extraction of small-scale power line images. This makes it possible to effectively learn the shallow edge features of power lines, even when the number of network layers is deeper. ResNet18 has a relatively shallow number of network layers, which is more advantageous to run on the equipment with limited memory under the premise of meeting the requirements of power line detection, and its backbone network structure is shown in Figure 1.

### 2.2. Enhanced Network Lightweighting Based on the Ghost Module

**Figure 2:**
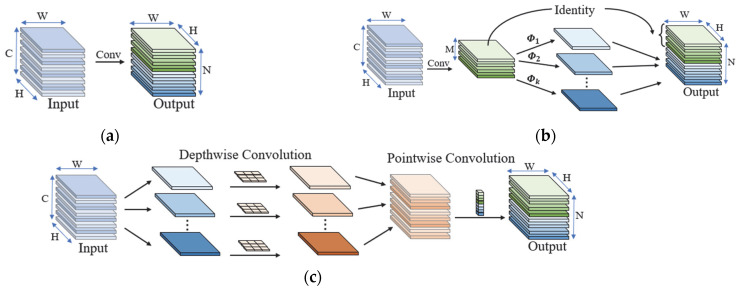
Diagram of three different convolution structures: (**a**) Ordinary Convolution; (**b**) Ghost Module; and (**c**) Depthwise Separable Convolution.

Although ResNet18 is constructed with a relatively modest stack of 18 conventional convolutional layers, its computational footprint can be further optimized through lightweight convolution to save storage space in edge devices. Among current lightweight convolution strategies, Depthwise Separable Convolution and Ghost Module [23] are widely adopted. Figure 2 shows the structural comparisons of ordinary convolution, Ghost Module, and Depthwise Separable Convolution, respectively.

The expressions for the ordinary convolution (Figure 2a) parametric quantity $P_{1}$ and computational quantity $S_{1}$ are:

(1) $$P_{1}=N\cdot C\cdot K^{2}$$

(2) $$S_{1}=N\cdot H\cdot W\cdot C\cdot K^{2}$$

where *C* denotes the number of input channels, *N* denotes the number of output feature map channels, *H* and *W* are the height and width of the output data, respectively, and K⋅K is the convolution kernel size.

In the Ghost Module (Figure 2b), M ordinary feature layers are generated by using a convolution operation on the feature layer. Then, the ordinary feature layer is subjected to linear operation Φ to produce redundant feature maps. Finally, the output is obtained by splicing the feature maps obtained in the first step with the Ghost feature maps obtained in the second step (identity), where the intermediate channel M=0.5N [24]. The Ghost Module parametric quantity $P_{2}$ and computational quantity $S_{2}$ are expressed as:

(3) $$P_{2}=0.5N\cdot C+0.5N\cdot K^{2}$$

(4) $$S_{2}=0.5N\cdot C\cdot H\cdot W+0.5N\cdot H\cdot W\cdot K^{2}$$

Depthwise Separable Convolution (Figure 2c) first performs channel-wise convolution, splices each channel after convolution, then performs pointwise convolution (1 × 1 convolution) on the spliced feature maps, and finally generates the feature output by weighting the feature maps from the previous step in the depth direction. The expressions for its parametric quantity $P_{3}$ and computational quantity $S_{3}$ are:

(5) $$P_{3}=N\cdot C+C\cdot K^{2}$$

(6) $$S_{3}=C\cdot H\cdot W\cdot K^{2}+N\cdot C\cdot H\cdot W$$

If the Ghost Module is used instead of the traditional convolution, the number of theoretical parameters and the computational comparison are:

(7) $$R_{p_{1}}=\frac{0.5N\cdot C+0.5N\cdot K^{2}}{N\cdot C\cdot K^{2}}=\frac{1}{2C}+\frac{1}{2K^{2}}$$

(8) $$R_{s_{1}}=\frac{0.5N\cdot C\cdot H\cdot W}{N\cdot H\cdot W\cdot C\cdot K^{2}}+\frac{0.5N\cdot H\cdot W\cdot K^{2}}{N\cdot H\cdot W\cdot C\cdot K^{2}}=\frac{1}{2C}+\frac{1}{2K^{2}}$$

If the depth-separable convolution is used instead of the conventional convolution, the theoretical parametric and computational quantities are compared as:

(9) $$R_{p_{2}}=\frac{N\cdot C+C\cdot K^{2}}{N\cdot C\cdot K^{2}}=\frac{1}{N}+\frac{1}{K^{2}}$$

(10) $$R_{s_{2}}=\frac{C\cdot H\cdot W\cdot K^{2}+N\cdot C\cdot H\cdot W}{N\cdot H\cdot W\cdot C\cdot K^{2}}=\frac{1}{N}+\frac{1}{K^{2}}$$

where the number of input channels *C* and the number of output feature map channels are usually very large and can be introduced by Equations (8) and (10):

(11) $$\frac{1}{2C}+\frac{1}{2K^{2}}<\frac{1}{N}+\frac{1}{K^{2}}$$

As shown in Equation (11), when outputting the same channel, the Ghost Module demonstrates the lowest computational cost and parameter count. By incorporating Ghost-assisted convolutions, the model is enabled to learn features at diverse scales and abstraction levels, thereby enhancing its representational capacity. Therefore, in this paper, we replace the standard convolutions within the residual blocks of ResNet18 with Ghost Modules for optimization.

### 2.3. Class Residuals Embedding Attention Mechanisms Across Layers

Attention mechanisms are incorporated into neural networks to emulate the human cognitive process of attention and selective information processing. Attention mechanisms enable the neural network to dynamically select and focus on different parts of the input data, thus improving the expressive power and performance of the model.

**Figure 3:**
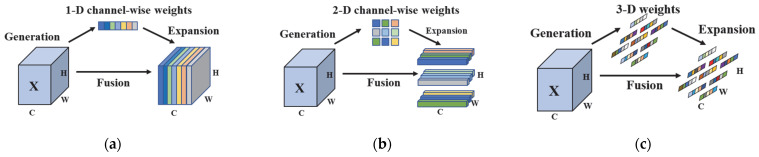
Comparison of different attention mechanisms: (**a**) channel attention mechanism; (**b**) spatial attention mechanism; and (**c**) 3D attention mechanism SIMAM.

Currently, mainstream attention mechanisms are divided into channel attention mechanisms and spatial attention mechanisms, of which ECA [25] (efficient channel attention) and CA [26] (channel attention) are typical representatives of channel and spatial attention mechanisms, respectively. The channel attention mechanism ECA (Figure 3a) improves the model feature representation from the perspective of one-dimensional information acquisition by assigning different weights to each channel to help the model automatically learn and emphasize the important feature channels in the convolutional layer. The spatial attention mechanism CA (Figure 3b), on the other hand, from the perspective of two-dimensional information acquisition, can capture both direction and position sensing information to strengthen the network feature extraction capability.

However, the human brain integrates channel and spatial attention in a collaborative manner, whereas ECA and CA can only refine features along single dimensions (channel or spatial). Their reliance on one-dimensional or two-dimensional weighting for cross-channel spatial information extraction limits the model’s ability to learn joint channel–spatial weights. In contrast, the attention mechanism SIMAM [27] (Figure 3c) creates 3D weight modules (incorporating both spatial and channel dimensions) to evaluate the importance of each neuron, which allows the network to further enhance the extraction of the target features by considering both space and channel, suppressing the redundant information and accomplishing this without increasing the number of any network parameter.

Therefore, in this paper, the attention mechanism SIMAM is chosen to be embedded in the codec of the network. SIMAM defines the following energy function for each neuron to weigh the weights between neurons:

(12) $$e_{t}(w_{t,}b_{t},y,x_{i})={\left(yt-\overset{⌢}{t}\right)}^{2}+\frac{1}{M-1}\sum_{i=1}^{M-1}{\left(y_{0}-{\overset{⌢}{x}}_{i}\right)}^{2}$$

where $t=w_{t}t+b_{t}$ and $x_{i}=w_{t}x_{i}+b_{t}$ are linear transformations of t and $x_{i}$ and t and $x_{i}$ are the target neuron and other neurons in a single channel of input feature $X\in R^{C\times H\times W}$. i is an index in the spatial dimension, and M=H×W is the number of neurons on that channel. $w_{t}$ and $b_{t}$ are weights and biases on the transform. The solution is obtained by iteratively solving:

(13) $$w_{t}=-\frac{2(t-\mu_{t})}{{\left(t-\mu_{t}\right)}^{2}+2\sigma_{t}^{2}+2\lambda },b_{t}=-\frac{1}{2}(t+\mu_{t})w_{t}$$

Assuming that all pixels in a single channel follow the same distribution, the mean and variance on all neurons can be calculated. Therefore, the minimum energy is:

(14) $$e_{t}^{*}=\frac{4({\overset{⌢}{\sigma }}^{2}+\lambda )}{{\left(t-\overset{⌢}{\mu }\right)}^{2}+2\sigma_{t}^{2}+2\lambda }$$

Equation (14) shows that the lower the energy, the more distinct the neuron is from the peripheral neurons and the more important it is for visual processing. The SIMAM module is refined as follows:

(15) $$\tilde{X}=sigmoid(\frac{1}{E})⊙X$$

**Figure 4:**
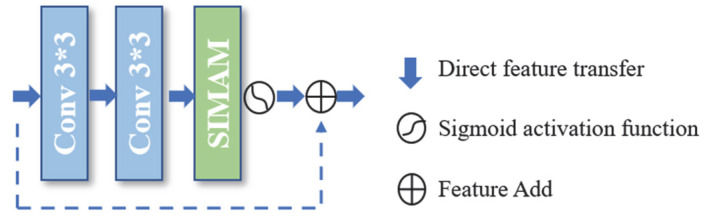
Attentional mechanisms of the SIMAM class in the residual additive approach.

The integration approach of attention mechanisms into neural networks significantly impacts model detection accuracy. In this paper, we systematically investigate the embedding strategy of the SIMAM module within the backbone network. Instead of directly inserting the attention module, we propose a novel residual attention fusion method (Figure 4). After the attention mechanism, the activation function sigmoid is accessed, and the feature information of the input of the previous layer is fused with the output of the embedded attention mechanism through the “short wiring” for cross-layer feature fusion. Through the short wiring connection, the features of the previous layers can be directly transferred to the latter layers, which provides more semantic information for the feature extraction of the model, improves the reuse rate of the features, and further enhances the network’s extraction of the features of the power lines in the complex background.

### 2.4. Enhancing the Robustness of Networks Based on Activation Functions

**Figure 5:**
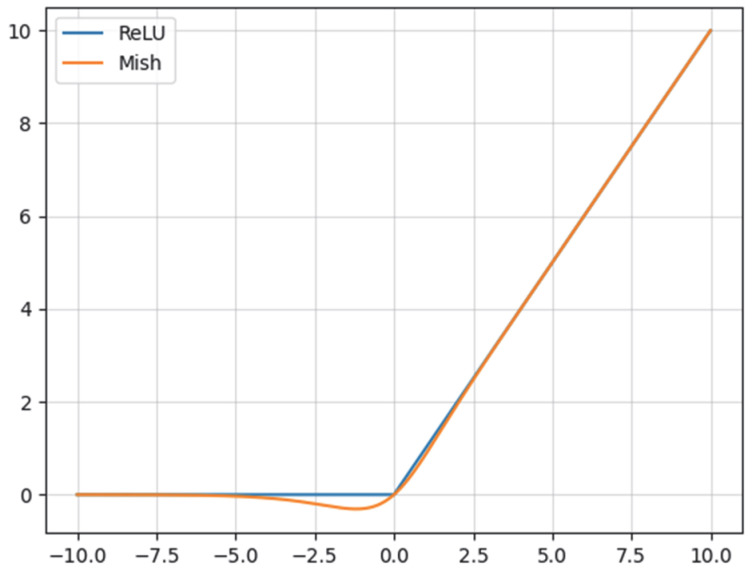
Plot of the Mish activation function versus ReLU activation function.

Activation functions serve as pointwise nonlinear transformations that introduce nonlinearity into neural network layers, governing how input signals are aggregated and activated. The ReLU activation function used in ResNet18 residual blocks is illustrated in Figure 5. The ReLU function exhibits a gradient of 1 in the positive domain and 0 in the negative domain. During backpropagation, the zero gradient in the negative domain can cause gradient extinction during model training, slowing the weight update of underlying neurons and impeding model convergence. Additionally, the non-differentiability of the ReLU function at the input value of 0 leads to gradient computation failures in backpropagation, further affecting network parameter updates and training efficiency.

Mish [28] is a non-monotonic, smooth, and self-regularizing activation function. As shown in Figure 5, compared with ReLU, Mish exhibits smoothness around the zero point, which mitigates the gradient vanishing issue, Mish upper unboundedness avoids data saturation, and lower boundedness enhances the function regularization, which prevents the neural network from overfitting to a certain extent and improves overall robustness. Consequently, this paper reconstructs the ReLU activation function in ResNet18 residual blocks with the Mish activation function.

### 2.5. RGS-UNet Model

**Figure 6:**
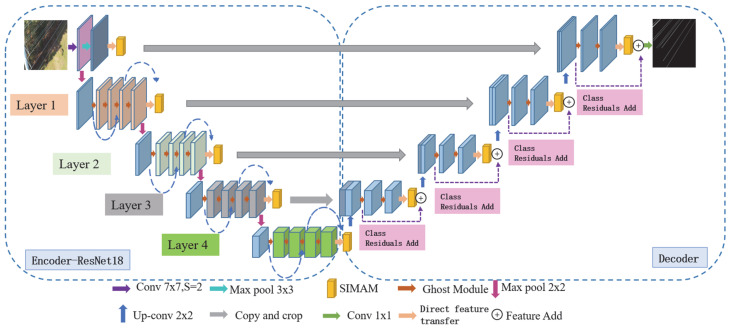
Model RGS-UNet structure diagram.

In summary, the improved model is designated as RGS-UNet (ResNet18-Ghost Module-SIMAM-UNet), and its architectural block diagram is presented in Figure 6.

## 3. Experimental Results and Analysis

### 3.1. Dataset and Experimental Environment

**Figure 7:**
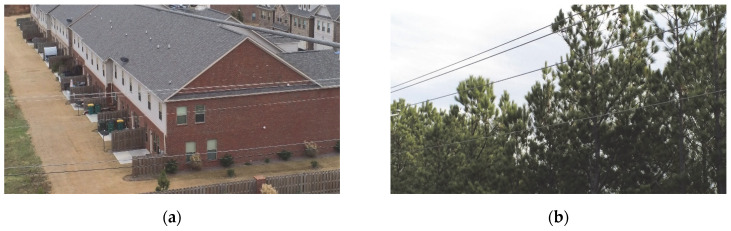

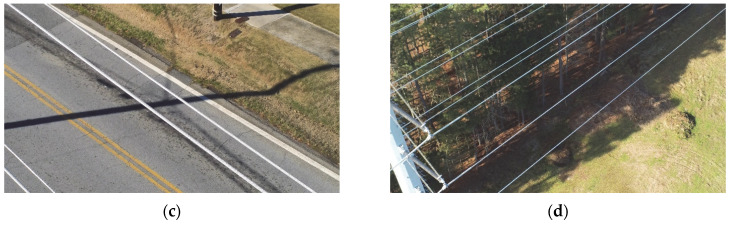
Sample of selected datasets shows the power lines images in various environment: (**a**) buildings; (**b**) woods; (**c**) roads; (**d**) grass.

The dataset employed in this paper is sourced from UAV field operations collected by the State Grid Corporation (SGC), comprising 1040 power line images with a resolution of 3840 × 2160 pixels. These images were annotated using LabelMe (v5.6.1) software, and the dataset was augmented to 2038 images through techniques such as image padding, rotation, scaling, and adjustments to brightness/contrast. As illustrated in Figure 7, power lines occupy a relatively small portion of the image resolution, with their shapes closely resembling background elements like building contours and road lines. Additionally, the backgrounds of these images exhibit significant diversity and complexity. During model training, the dataset was randomly partitioned into training, validation, and test sets at an 8:1:1 ratio.

All experiments in this paper were conducted in the Ubuntu 18.08 system; the deep learning framework used was PyTorch 1.8.0, Python 3.7.9, CUDA = 11.2, and the training configuration was an RTX A6000/48G graphics card (NVIDIA, Santa Clara, CA, USA).

### 3.2. Experimental Procedure

In the experimental training process of this paper, the training idea of migration learning is adopted for the model, and the training is divided into two stages. The first stage involves 50 rounds of freezing iterations, while the second stage consists of 160 rounds of unfreezing training. The initial learning rate during the model’s training period is set to 1 × 10^−4^, the batch size is 4, and the Adam optimization strategy is employed.

**Figure 8:**
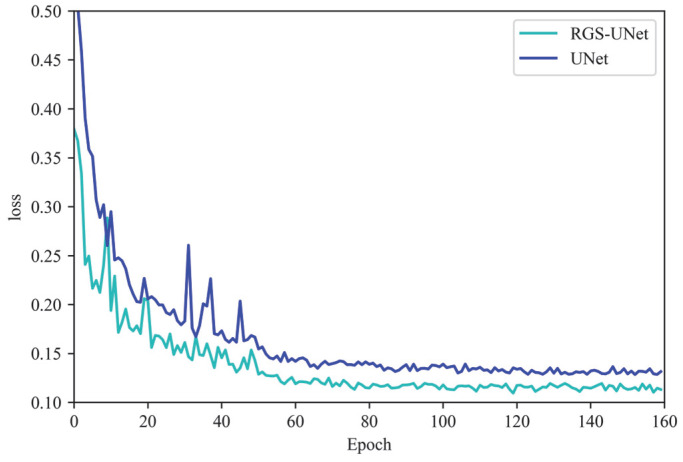
Loss curve diagram.

Figure 8 presents the training loss curves for the original UNet model and the improved RGS-UNet model. As illustrated in Figure 8, during the initial phase of training, the loss value decreases rapidly for both models. This rapid decline indicates that the models can quickly learn from the initial state. As the number of iterations increases, the loss function curves enter a period of smoothness, where the loss values exhibit minimal change. This plateau signifies that the models have converged and stabilized. Notably, the improved RGS-UNet model demonstrates significantly smaller loss fluctuations and lower loss values compared with the original UNet model. These characteristics suggest that the RGS-UNet model has superior performance and fitting ability.

### 3.3. Experimental Evaluation and Analysis of Results

To validate the effectiveness of the improvement strategy proposed in this paper, we employed three key evaluation criteria for segmentation accuracy: the F1-Score [29], intersection over union (IoU) [30], and parameter number (Params).

F1-Score is a metric for evaluating binary classification models, defined as the weighted harmonic mean of precision and recall. A higher F1-Score indicates superior model performance.

(16) $$F1-Score=\frac{2precision\times recall}{precision+recall}=\frac{2TP}{2TP+FP+FN}$$

IoU evaluates a model’s pixelwise classification accuracy by measuring the overlap between predicted segmentation results and ground truth masks. A higher IoU value indicates more precise segmentation performance.

(17) $$IoU=\frac{TP}{TP+FP+FN}$$

where *TP* (true positive) indicates that pixels labelled as wires are correctly identified, *FN* (false negative) indicates that pixels labelled as wires are incorrectly identified as background, *TN* (true negative) indicates that pixels labelled as background are correctly identified, and *FP* (false positive) indicates that pixels labelled as background are incorrectly identified as wires.

To verify that multiple improvement strategies all improve the performance of power line segmentation, this paper trains multiple sets of network models for comparative experiments.

(1) Comparison Based on Different Backbone Networks

PP-LCNet, MobileNetV3, GhostNet v2, FasternetT2, RepVGG, EfficientNetV2, and ResNet18 are used as the backbone network, respectively, and the experimental results are shown in Table 1.

As shown in Table 1, the backbone network ResNet18 adopted in this paper achieves higher F1-Score and IoU values than other backbone networks. Compared with baseline UNet, its F1-Score and IoU are improved by 1.2% and 1.47%, respectively, while the parameter count is compressed to 79.55% of the original.

(2) Experimental Comparison of Introducing Different Attention Mechanisms and Different Embedding Methods

The experimental models in this group are based on the ResNet18 backbone, where standard convolutions in residual blocks are optimized to Ghost Modules. Different attention mechanisms—ECA, CA, and SIMAM—are embedded, respectively, and the class residual embedding method is experimentally compared against the conventional embedding approach. The results are presented in Table 2.

The experimental data comparison in Table 2 shows that: (1) the F1-Score and IoU of the 3D-weighted SIMAM attention mechanism are higher than those of CA and ECA with the attention mechanism that focuses only on the channel or space; (2) a two-by-two comparison of ECA and ECA+ class residuals, CA and CA+ class residuals, SIMAM and SIMAM+ class residuals reveals that all the F1-Score and IoU values of the models using the 3D-weighted class residuals method to embed the attention mechanism have been further improved compared with the models without this method to introduce the attention mechanism; and (3) using class residuals to embed the SIMAM attention mechanism is most beneficial, achieving an F1-score of 90.83% and an IoU value of 84.89%, respectively, without introducing any additional parameters.

(3) Ablation Experiment

The third experiment is set up to verify the effectiveness of multiple improvement strategies, and an ablation experiment is set up, where the symbol “√” denotes the inclusion of a module. The improved model RGS-UNet is compared with the methods in the literature [15,16], and the experimental results are presented in Table 3.

As shown in Table 3, replacing the standard convolution in ResNet18 residual blocks with Ghost Modules—while using ResNet18 as the UNet backbone—reduces model parameters to 57.25% of the original UNet, achieving significant lightweighting. Embedding the SIMAM attention mechanism via a residual-like approach improves F1-Score by 1.67% and IoU by 2.15% over the baseline UNet without increasing parameters, enhancing power line feature extraction ability. Substituting ReLU with the Mish activation function in deep convolutions further boosts F1-Score by 1.54% and IoU by 1.95%. When combining all improvements, the fully optimized RGS-UNet achieves 2.05% and 2.58% gains in F1-Score and IoU, respectively, compared with the original UNet. Additionally, RGS-UNet outperforms the methods in References [15,16] in accuracy, though further model size optimization is still warranted.

### 3.4. Comparison of Overall Detection Results of the Improved Model

**Figure 9:**
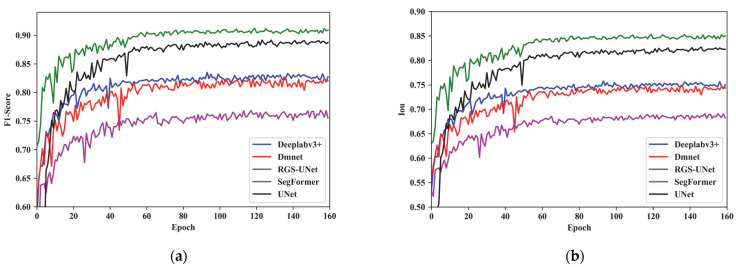
Comparison curves of F1-Score and IoU for each model: (**a**) comparison curves of F1-Scores; (**b**) comparison curves of IoU.

To validate the practical detection performance of the improved model, this paper conducts comparative experiments among RGS-UNet, UNet, DMNet, DeepLabv3+, and SegFormer. Figure 9 illustrates the F1-Score and IoU comparison curves for each model. As evident in Figure 9a,b, the improved model demonstrates significantly superior detection accuracy compared with other models.

**Figure 10:**
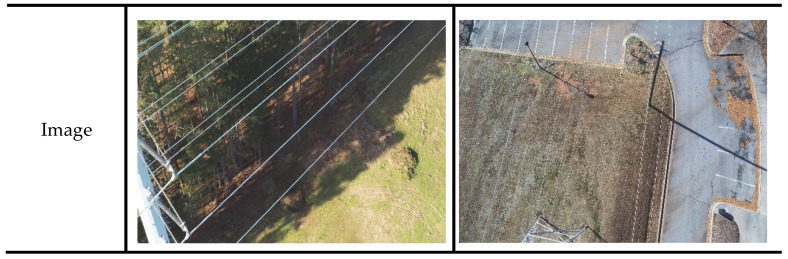

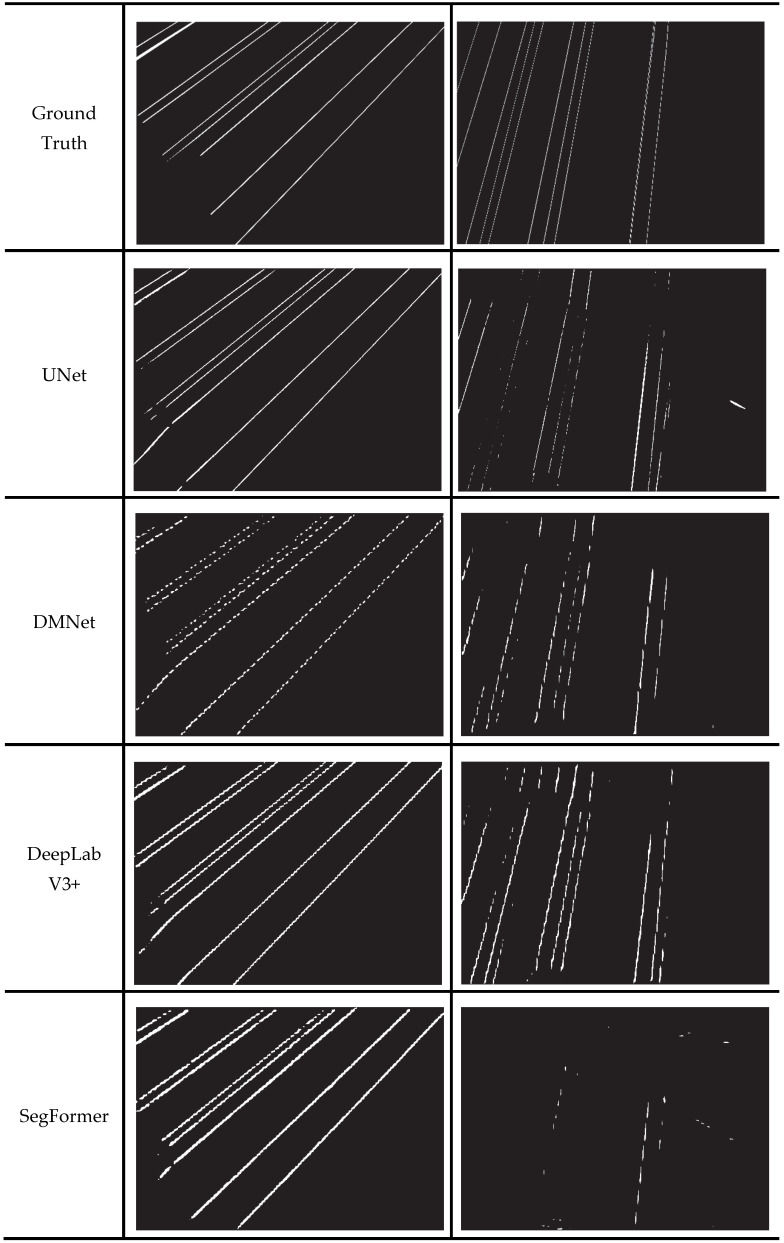

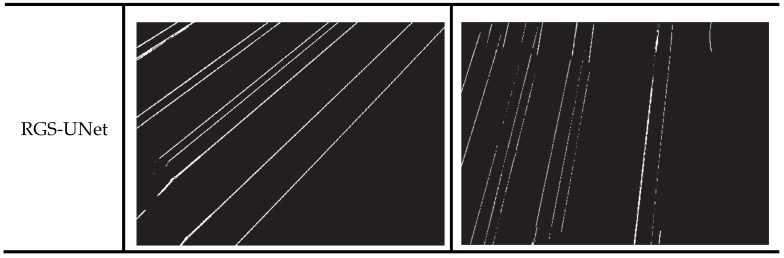
Comparison curves of F1-Score and IoU for each model.

Sample power line segmentation results from the test set are displayed in Figure 10. The first row shows aerial photography real images, while the second row presents ground truth images—manually labeled via LabelMe to annotate targets, primarily used for evaluating and comparing predicted images during the training process.

As shown in Figure 10, current network models exhibit significant variations in environmental sensitivity when performing power line detection tasks. In simple backgrounds with high contrast, all models can accurately identify power line targets. However, in complex backgrounds—particularly when power lines are adjacent to or overlapping with background lines—UNet, DMNet, DeepLab V3+, and SegFormer show severe issues such as discontinuous detections, false positives, and false negatives despite roughly outlining the location and contour of power lines. By comparison, RGS-UNet demonstrates far fewer issues of this type and achieves the most superior detection performance among all models.

**Figure 11:**
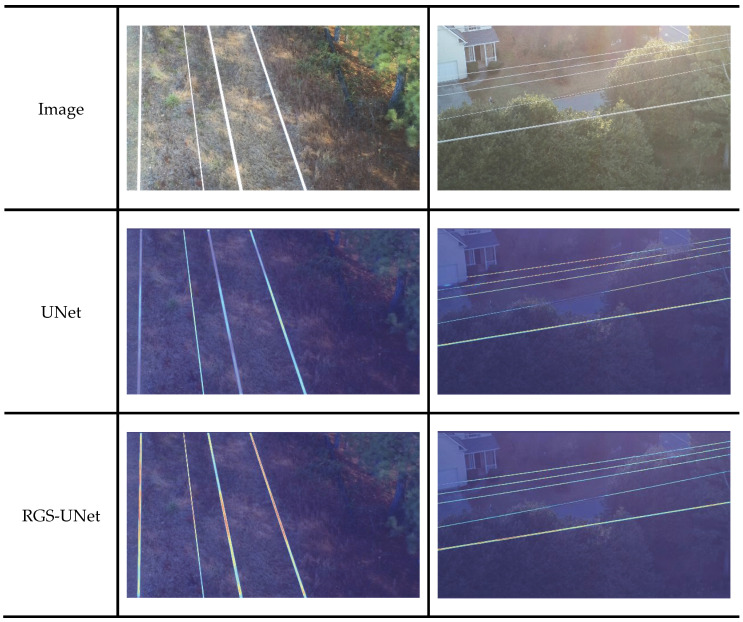
Comparison of Grad-CAM heatmap visualization results.

To intuitively compare the performance disparities between the original UNet and the improved RGS-UNet on real images, this paper analyzes their prediction result heatmaps on actual power line photographs. This approach enables a more accurate evaluation of the improved model’s practical effectiveness. The feature focus of UNet and its improved version RGS-UNet is analyzed via Grad-CAM heatmap visualization, with the detection results presented in Figure 11. Both UNet and RGS-UNet effectively respond to the main power line area and accurately identify power lines. However, RGS-UNet exhibits a larger heatmap region and darker target color compared with UNet, indicating that it assigns higher weights to the targets during power line identification. This leads to more precise power line detection accuracy.

### 3.5. Edge Device Deployment

The original UNet and the improved RGS-UNet are deployed on edge devices for testing to comprehensively validate the algorithms’ performance in practical application scenarios. Jetson Xavier NX (NVIDIA, Santa Clara, CA, USA) is selected as the target edge device for porting due to its rich interfaces that enable seamless integration with various external devices. It not only delivers exceptional computational capabilities but also features low power consumption and a compact design, establishing itself as an ideal platform for developing and deploying applications on hardware-constrained devices such as UAVs. During testing, its integrated hardware camera captures real-time data, providing authentic and diverse datasets for algorithm validation, thereby ensuring the validity and reliability of test results. The detailed technical specifications of Jetson Xavier NX are presented in Table 4. The original UNet and the improved RGS-UNet are deployed on Jetson Xavier NX, respectively, and their detection speeds are compared in Table 5.

As shown in Table 5, the proposed RGS-UNet achieves a detection accuracy of 91.21% F1-Score, representing a 2.05% improvement over the baseline UNet. This indicates that RGS-UNet enables more accurate target recognition while reducing the false positive rate and false negative rate. In terms of detection speed, RGS-UNet reduces per-frame inference time from 0.58 s to 0.39 s, significantly enhancing the efficiency and smoothness of the detection system when processing continuous image streams.

**Figure 12:**
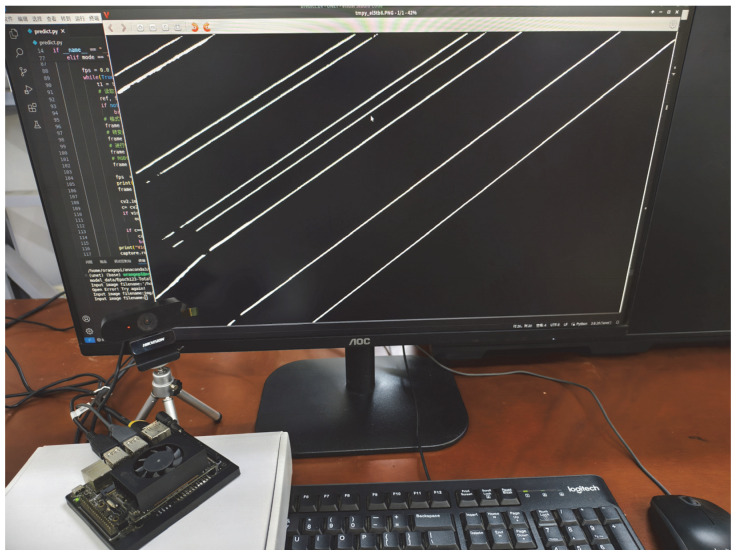
Algorithm deployment of Jetson Xavier NX.

Figure 12 visually demonstrates the actual operation of the RGS-UNet model on Jetson Xavier NX. It shows that RGS-UNet maintains robust detection performance even in the resource-constrained environment, accurately segmenting power lines with high precision. This demonstrates the model’s strong robustness and accuracy in practical applications. Additionally, the embedded device tested in this study is well suited for UAVs deployment, effectively adapting to diverse hardware environments and real-world scenario requirements.

## 4. Conclusions

In this paper, a power line segmentation model RGS-UNet based on a lightweight network and residual-like cross-layer feature fusion module is proposed to address the challenges of large parameter counts and low segmentation accuracy in existing power line segmentation models.

RGS-UNet employs UNet as its backbone and adopts ResNet18 as the feature extraction network, effectively enhancing semantic information extraction for small-size images. By replacing standard convolutions in ResNet18’s residual blocks with Ghost Modules, the model significantly reduces computational load. Meanwhile, the SIMAM attention mechanism—embedded in the encoder–decoder stage via a residual-style architecture—enables the network to focus on power line features in complex backgrounds, suppressing irrelevant information. The Mish activation function is applied in deep convolutional layers to mitigate gradient vanishing and improve network robustness. Finally, the model is evaluated on UAV-based power line detection datasets and compared with state-of-the-art semantic segmentation models. Experimental results show that compared with the classical UNet, the optimized network achieves 2.05% and 2.58% improvements in F1-Score and IoU, respectively, while reducing the parameter count to 57.25% of the original model. RGS-UNet effectively addresses the challenge of balancing detection accuracy and model size, demonstrating that the proposed improvements significantly enhance power line segmentation performance and provide a valuable reference for practical applications.

However, several limitations warrant further improvement and optimization. Future research may delve into the following directions:

(1) Aiming at the challenges of high data labeling costs and weak cross-domain generalization in power line semantic segmentation, domain adaptation techniques or self-supervised pre-training via generative adversarial networks could be explored. These may reduce reliance on large-scale labeled datasets and enhance model migration capabilities.

(2) Integrating visible light data with multi-source modalities—such as infrared, ultraviolet, satellite remote sensing, laser scanning, and inspection tracks—may foster more efficient and comprehensive inspection outcomes.

## Figures and Tables

**Table 1:** Experimental results of different backbone networks.

Model	F1-Score (%)	IoU (%)	Params/MB
UNet	89.16	82.74	24.89
PP-LCNet	87.54	80.68	16.48
MobileNetV3	86.34	79.10	8.48
GhostNet v2	87.39	80.33	9.56
FasternetT2	82.97	75.12	25.64
RepVGG	83.86	76.38	19.60
EfficientNetV2	84.91	77.68	26.67
ResNet18	90.36	84.21	19.80

**Table 2:** Comparison of attention mechanism integration.

Model	F1-Score (%)	IoU (%)	Params/MB
UNet	89.16	82.74	24.89
ECA	90.27	84.10	14.25
ECA+ Class Residuals	90.52	84.39	14.25
CA	90.33	84.17	14.31
CA+ Class Residuals	90.61	84.57	14.31
SIMAM	90.57	84.55	14.25
SIMAM+ Class Residuals	90.83	84.89	14.25

**Table 3:** Results of ablation experiments.

Method	ResNet	Ghost Module	SIMAM	Mish	F1-Score (%)	IoU (%)	Params/MB	FLOPs (G)
UNet					89.16	82.74	24.89	451.67
Improvement 1	√				90.36	84.21	19.80	334.64
Improvement 2	√	√			90.25	84.14	14.25	299.67
Improvement 3	√	√	√		90.83	84.89	14.25	299.67
Improvement 4	√	√		√	90.70	84.69	14.25	299.67
RGS-UNet	√	√	√	√	91.21	85.32	14.25	299.67
Y-UNet [15]					87.05	80.13	3.97	-
G-UNet [16]					89.24	82.98	2.99	-

**Table 4:** NVIDIA Jetson Xavier NX technical parameter sheet.

Name	Technical Parameters
CPU	6-core NVIDIA Carmel ARM^®^v8.2 64-bit
GPU	384-core NVIDIA Volta TM GPU 48 Tensor Cores (21TOPS)
RAM	8 GB 128-bit LPDDR4x 51.2 GB/s
Memory	16 GB eMMC5.1
Network	1000 BASE-T Ethernet
Power Wastage	10 W/15 W

**Table 5:** Comparison of networks with and without the Mish activation function.

Model	F1-Score (%)	Speed (s)	Params/MB
UNet	89.16	0.58	24.89
RGS-UNet	91.21	0.39	14.25

## Data Availability

The original contributions presented in this study are included in the article. Further inquiries can be directed to the corresponding author.

## References

[1] Zhu J., Guo Y., Yue F., Yuan H., Yang A., Wang X., Rong M. (2020). A Deep Learning Method to Detect Foreign Objects for Inspecting Power Transmission Lines. IEEE Access.

[2] Chen M.L., Wang Y.Z., Dai Y., Yan Y.F., Qi D.L. (2022). SaSnet: A real-time power line segmentation network based on self-supervised learning. Proc. CSEE.

[3] Wang L., Chen Z., Hua D., Zheng Z. (2019). Semantic Segmentation of Transmission Lines and Their Accessories Based on UAV-Taken Images. IEEE Access.

[4] Wei S.X., Li Y., Shuang F., Zhou Z., Li P., Li Z. (2024). Power Line Extraction Algorithm for UAV Inspection Scene Images. Comput. Integr. Manuf. Syst..

[5] Zhou W., Ji C., Fang M. (2024). Effective Dual-Feature Fusion Network for Transmission Line Detection. IEEE Sens. J..

[6] Zhang C.X., Zhao L., Wang X.P. (2018). Fast Extraction Algorithm for Power Lines in Complex Feature Backgrounds. Eng. J. Wuhan Univ..

[7] Rong S., He L., Du L., Li Z., Yu S. (2021). Intelligent Detection of Vegetation Encroachment of Power Lines with Advanced Stereovision. IEEE Trans. Power Deliv..

[8] Shuang F., Chen X., Li Y., Wang Y., Miao N., Zhou Z. (2022). PLE: Power Line Extraction Algorithm for UAV-Based Power Inspection. IEEE Sens. J..

[9] Chen X.Y., Xia J., Du K. (2021). Overhead transmission line detection based on multilinear feature enhancement network. J. Zhejiang Univ. (Eng. Sci.).

[10] Zhang Y.P., Wang W.H., Zhao S.P., Zhao S.X. (2022). Research on automatic extraction of railroad contact network power lines in complex background based on RBCT algorithm. High Volt. Eng..

[11] Zhao L., Wang X.P., Yao H.T., Tian M. (2021). A Review of Power Line Extraction Algorithms Based on Visible Light Aerial Images. High Volt. Eng..

[12] Yang L., Kong S., Deng J., Li H., Liu Y. (2023). DRA-Net: A Dual-Branch Residual Attention Network for Pixelwise Power Line Detection. IEEE Trans. Instrum. Meas..

[13] Yang L., Kong S., Cui S., Huang H., Liu Y. An Efficient End-to-End CNN Network for High-voltage Transmission Line Segmentation. Proceedings of the 2022 IEEE 8th International Conference on Cloud Computing and Intelligent Systems (CCIS).

[14] Liu J.W., Li Y.X., Gong Z., Liu X.G., Zhou Y.J. (2020). Full Convolutional Network Wire Recognition Method. J. Image Graph..

[15] Yang L., Fan J., Xu S., Li E., Liu Y. (2022). Vision-Based Power Line Segmentation with an Attention Fusion Network. IEEE Sens. J..

[16] Han G., Zhang M., Li Q., Liu X., Li T., Zhao L., Liu K., Qin L. (2022). A Lightweight Aerial Power Line Segmentation Algorithm Based on Attention Mechanism. Machines.

[17] Liu J.-J., Hou Q., Liu Z.-A., Cheng M.-M. (2023). PoolNet+: Exploring the Potential of Pooling for Salient Object Detection. IEEE Trans. Pattern Anal. Mach. Intell..

[18] Cui C., Gao T., Wei S., Du Y., Guo R., Dong S., Lu B., Zhou Y., Lv X., Liu Q. (2021). PP-LCNet: A Lightweight CPU Convolutional Neural Network. arXiv.

[19] Huang L., Xiang Z., Yun J., Sun Y., Liu Y., Jiang D., Ma H., Yu H. (2023). Target Detection Based on Two-Stream Convolution Neural Network with Self-Powered Sensors Information. IEEE Sens. J..

[20] Tang Y., Han K., Guo J., Xu C., Xu C., Wang Y. (2022). GhostNetV2: Enhance Cheap Operation with Long-Range Attention. Adv. Neural Inf. Process. Syst..

[21] El Ariss O., Hu K. (2023). ResNet-Based Parkinson’s Disease Classification. IEEE Trans. Artif. Intell..

[22] Shafiq M., Gu Z. (2022). Deep Residual Learning for Image Recognition: A Survey. Appl. Sci..

[23] He Z., He D., Li X., Qu R. (2023). Blind Superresolution of Satellite Videos by Ghost Module-Based Convolutional Networks. IEEE Trans. Geosci. Remote Sens..

[24] Zhang R.H., Ou J.S., Li X.M., Ling X., Zhu Z., Hou B.F. (2023). Lightweight pineapple seedling heart detection algorithm based on improved YOLOv4. Trans. Chin. Soc. Agric. Eng..

[25] Wang Q., Wu B., Zhu P., Li P., Zuo W., Hu Q. ECA-Net: Efficient Channel Attention for Deep Convolutional Neural Networks. Proceedings of the 2020 IEEE/CVF Conference on Computer Vision and Pattern Recognition (CVPR).

[26] Hou Q., Zhou D., Feng J. (2021). Coordinate Attention for Efficient Mobile Network Design. arXiv.

[27] Yang L., Zhang R.-Y., Li L., Xie X. SimAM: A Simple, Parameter-Free Attention Module for Convolutional Neural Networks. Proceedings of the 38th International Conference on Machine Learning.

[28] Lu Y.-F., Gao J.-W., Yu Q., Li Y., Lv Y.-S., Qiao H. (2023). A Cross-Scale and Illumination Invariance-Based Model for Robust Object Detection in Traffic Surveillance Scenarios. IEEE Trans. Intell. Transp. Syst..

[29] Wang B., Yang K., Zhao Y., Long T., Li X. (2023). Prototype-Based Intent Perception. IEEE Trans. Multimed..

[30] Chang H., Fu X., Guo K., Dong J., Guan J., Liu C. (2025). SOLSTM: Multisource Information Fusion Semantic Segmentation Network Based on SAR-OPT Matching Attention and Long Short-Term Memory Network. IEEE Geosci. Remote Sens. Lett..

